# Intratumoral Administration of a Novel Cytotoxic Formulation with Strong Tissue Dispersive Properties Regresses Tumor Growth and Elicits Systemic Adaptive Immunity in In Vivo Models

**DOI:** 10.3390/ijms21124493

**Published:** 2020-06-24

**Authors:** Lewis H. Bender, Franco Abbate, Ian B. Walters

**Affiliations:** Intensity Therapeutics, Inc., Westport, CT 06880, USA; fabbate@intensitytherapeutics.com (F.A.); iwalters@intensitytherapeutics.com (I.B.W.)

**Keywords:** intratumoral, INT230-6, dispersion, diffusion, cytotoxic, cisplatin, vinblastine, cell-penetration, enhancer, immune activation, PD-1, antibody, CTLA-4, checkpoint

## Abstract

The recent development of immune-based therapies has improved the outcome for cancer patients; however, adjuvant therapies remain an important line of treatment for several cancer types. To maximize efficacy, checkpoint inhibitors are often combined with cytotoxic agents. While this approach often leads to increased tumor regression, higher off target toxicity often results in certain patients. This report describes a novel formulation comprising a unique amphiphilic molecule, 8-((2-hydroxybenzoyl)amino)octanoate (SHAO), that non-covalently interacts with payloads to increase drug dispersion and diffusion when dosed intratumorally (IT) into solid tumors. SHAO is co-formulated with cisplatin and vinblastine (referred to as INT230-6). IT dosing of the novel formulation achieved greater tumor growth inhibition and improved survival in in vivo tumor models compared to the same drugs without enhancer given intravenously or IT. INT230-6 treatment increased immune infiltrating cells in injected tumors with 10% to 20% of the animals having complete responses and developing systemic immunity to the cancer. INT230-6 was also shown to be synergistic with programmed cell death protein 1 (PD-1) antibodies at improving survival and increasing complete responses. INT230-6 induced significant tumor necrosis potentially releasing antigens to induce the systemic immune-based anti-cancer attack. This research demonstrates a novel, local treatment approach for cancer that minimizes systemic toxicity while stimulating adaptive immunity.

## 1. Introduction

Cancer is both a local and systemic disease. The mainstay of treatment of many metastatic solid malignancies has been regional, i.e., surgery, radiation, ablation, with or without systemic anticancer agents given intravenously (IV) or orally [1]. Only small amounts of systemically administered anti-cancer drugs reach the vascular areas of the tumor with less drug reaching cancer cells in a tumor’s hypoxic regions [2]. The result of systemic dosing is low mass transfer into cancer cells, potentially incomplete dispersion throughout the tumors and poor patient outcomes. These challenges are more pronounced in larger tumors and metastatic disease. Additionally, certain genetic factors of cancer cells, such as those that limit the expression of internalizing drug-transport receptors or that increase efflux pump drug removal, can further reduce intracellular drug concentrations [3]. These factors contribute to the limited efficacy of systemically administered treatments [4]. Systemic delivery also distributes a drug throughout the body, which often results in off-target toxicities [5,6,7].

Direct intratumoral (IT) drug therapy, which has been investigated over the past several decades [8], could theoretically allow for higher doses to reach the tumor without an increase in systemic toxicity. IT delivery was initially studied with chemotherapeutic agents [9,10] using formulations that attempted to enhance residence time in the tumor by the addition of gels, vasoconstrictors, and other retention agents [9,10,11,12,13,14,15,16,17]. However, these approaches failed to improve efficacy over systemic delivery. The disappointing performance of IT dosing historically may be due, in part, to poor drug dispersion throughout the tumor when dosed [18], a low transmembrane flux rate inherent with receptor transport [3,19,20], and the inability of localized therapies to address the systemic nature of cancer (i.e., the circulating tumor cells and unseen micrometastases) [1]. Few regional chemical-based treatments have therefore become the standard of care, although transarterial chemoembolization or the use of alcohol dosed into localized small liver tumors is used regularly.

More recently, treatment approaches have shifted from killing the cancer cell to stimulating immune cells. This shift to immune-oncology (IO) treatment has reopened the investigations into intratumoral approaches focusing on activating local immune response. Indeed, a novel genetically modified oncolytic viral-based immunotherapeutic, talimogene laherparepvec (T-Vec), has been approved for IT use [21] in cutaneous melanoma. The objective of this viral approach is to transfect the granulocyte-macrophage colony-stimulating factor gene into the tumor microenvironment to recruit a local inflammatory response that would promote a systemic immune response. While T-Vec is approved solely for local treatment of localized cutaneous melanoma, the drug has not been shown to improve overall survival or have any effect on distal metastases [22]. Other local treatment approaches also attempt to recruit the immune system cells into the local tumor microenvironment. Data on several other intratumorally-delivered agents such as STING agonists, RIG-1, and TLR9 have been presented at major cancer conferences [23]. When the immune system is engaged, even at the single tumor level, there is the potential that a local IT approach could extend beyond the treated tumor. Accordingly, there remains a continued unmet need for the development of direct IT therapies for solid tumors that provide high local efficacy coupled with nontoxic systemic effects.

An ideal formulation for IT delivery would allow the anticancer agent to freely disperse the drug throughout the entire tumor, preferentially enter cancer cells, sparing healthy normal cells, and reaching the intended target either on the cancer cell surface or inside the cell. Amphiphilic molecules are compounds that are soluble in lipids and water systems. Some of these amphiphilic molecules can also bind noncovalently to active drugs, thereby forming supramolecular complexes [24]. The agents have been used to facilitate protein absorption in the gut for systemic delivery following oral administration [24,25,26]. Such amphiphilic compounds act as delivery agents for the drugs into cells by a passive diffusion process [24,25,27,28,29,30]. These agents have properties that improve the aqueous and lipid solubility of therapeutics to enhance transmembrane cell penetration via a diffusion process without damage to the membrane [24,28].

Herein, we report experimental data using a novel formulation consisting of an amphiphilic compound, 8-((2-hydroxybenzoyl)amino)octanoate (also referred to as SHAO), co-formulated in water at a fixed ratio with two potent agents, cisplatin and vinblastine sulfate. The formulation being referred to as INT230-6. Evidence is described of tissue dispersion, improved tumor kill and attraction of cells for potential immune activation. The new formulation is superior at tumor regression and increasing survival compared to the same drugs at the same concentrations alone given IV or IT without the amphiphilic agent. INT230-6 is now being evaluated as a treatment for solid cancers at clinical sites in the United States and Canada (clinicaltrials.gov NCT03058289).

A desirable feature of INT230-6 is selectivity penetrating cancer cells over healthy ones. For a number of tissues, the cancer cell has substantially increased membrane fluidity compared to healthy cells [18,20,31,32]. An amphiphilic formulation such as INT230-6 with greater lipid solubility would have greater diffusivity through the more fluid cancer cell’s lipid bilayer vis-a-vis the same organ’s healthy cell membrane. Thus, the amphiphilic formulation lipid soluble INT230-6 being physically injected into a tumor has cancer-cell-targeting potential. Toxicology studies conducted as part of the regulatory development process indeed demonstrated that the potent agents in INT230-6 following injection to a normal liver do not harm hepatocytes. SHAO is mixed with and non-covalently bound to its target payloads [24,28], once such formulation is in a dilutive environment (i.e., in the blood or the water compartment of cells), the compound is diluted away from the active agent. IT dosing thus distributes the drug payload either into the desired cancer cell or systemically at subtoxic concentrations (depending on amount dosed) via absorption in the vascular compartment.

The anticancer activity of INT230-6 as described below appears to be related to initial cytoreduction, recruitment of dendritic cells to process antigens created from the tumor that lead to a reduction in non-treated tumor volumes. In addition, immunological response is observed to further eradicate cancer cells and protect the animals from re-challenge.

## 2. Results

### 2.1. Diffusion and Dispersion of Drugs Using Amphiphilic Molecules

Malkov [24] reported that modified amino acid, amphiphilic molecules achieve passive drug transport across a colon cancer cell monolayer model into the cells. However, use of the amphiphilic agents for drug dispersion throughout tumors and into tumor cancer cells has not been previously reported.

**Figure 1:**
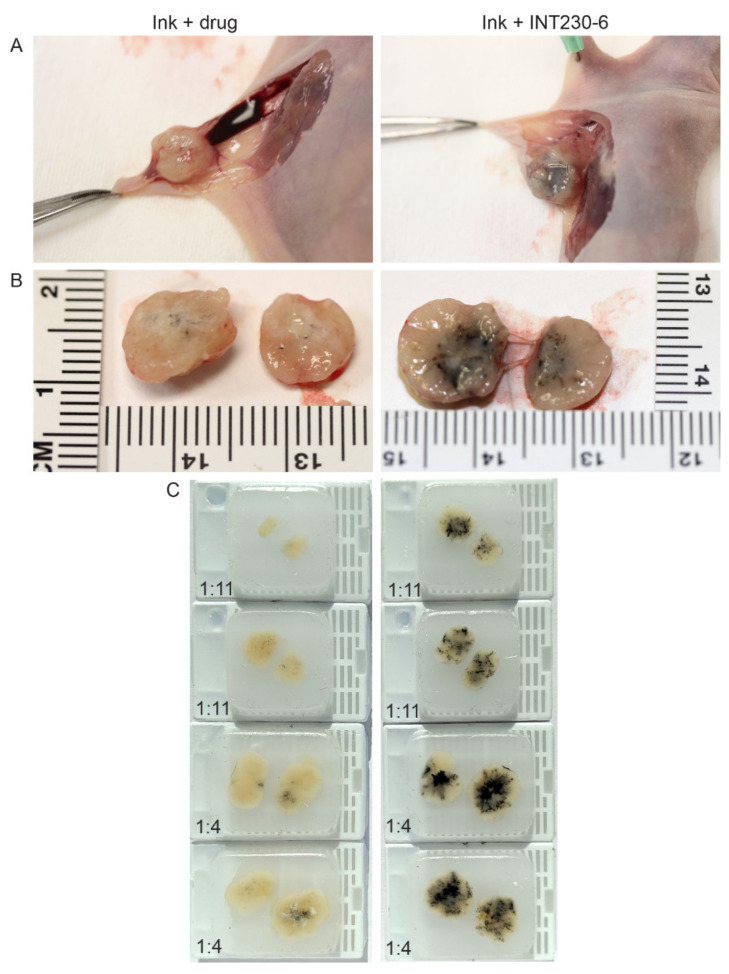
Comparison of dispersion of aqueous drug solutions containing -((2-hydroxybenzoyl)amino)octanoate (SHAO) with India Ink compared to aqueous vehicle with drug (cis) with Ink alone in BxPc3-luc2 pancreatic murine tumor xenografts. The images show unexcised (**A**) and excised tumors (**B**), bifurcated along the same plane, dosed with 0.075 mL (1:11) or 0.225 mL (1:4) of the INT230-6 formulation (which contains the enhancer) or drug control administered intratumorally over 90 s to >500-mm^3^ tumors. (**C**) Paraffin blocks were made from the injected tumors. Caliper measurements of the longest axis of the stained region were taken to estimate the degree of ink dispersion (INT230-6: mean 8.25 mm vs. drug alone: 2.8 mm *p* < 0.0002).

To test the dispersion of INT230-6 following direct intratumoral injection, formulations of INT230-6 combined with a non-colloidal ink or the ink plus the drug without the enhancer were injected intratumorally. The majority of the aqueous control drug-only solution was excreted into the surrounding interstitial space and not retained in the tumor (Figure 1A). INT230-6 with ink was fully absorbed into this dense tumor compared to the controls (Figure 1B). Upon excision and bisecting each tumor, paraffin blocks were made and utilized to quantify the ink dispersion (Figure 1C). The eight tumors receiving the enhancer-containing formulation had a significantly greater dispersion (as measured by the spread of ink in the bisected tumors) compared to controls (8.25 vs. 2.8 mm, *p* value < 0.0002). Visually, the spread of the solution throughout the tumor was much darker in the INT230-six injected tumors. The spread of the INT230-6 solution was dose dependent with the 1:4 ratio dispersing further with the tumor. The coloration of the drug alone in the tumors was also visually much lighter in color and showed little absorption or dispersion and was not dose to tumor ratio dependent. Additional diffusion experiments of this type were repeated at three different laboratories with similar results.

**Figure 2:**
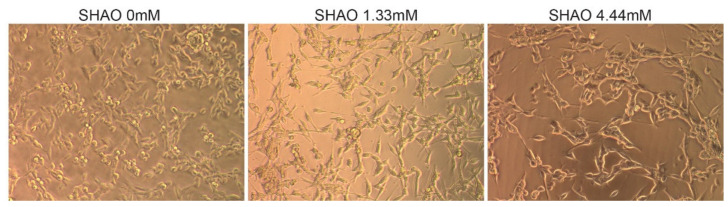
In vitro incubation showing cell morphology in the presence or absence of the SHAO molecule. Images showing 24 h of incubation in vitro of Colon-26 cells with SHAO: 0, 1.32 and 4.44 mM.

In addition to the in vivo experiment, SHAO was incubated in vitro, with 2 × 10^4^ cells per well at concentrations of 1.3 and 4.4 mM (Figure 2). The treatment did not destroy the cell membrane, even at 24 h of incubation time. When compared to the control cells, SHAO appeared to only have a minor concentration-dependent effect on cellular morphology.

Overall, these data show that the enhancer formulation appears to enable better diffusion and dispersion of the drugs throughout the tumors when all the compounds are administered intratumorally, as shown by the larger tumor regions stained by Ink in the presence of SHAO.

### 2.2. Tumor Growth Inhibition and Survival in Colon 26 Tumor Mouse Model

**Figure 3:**
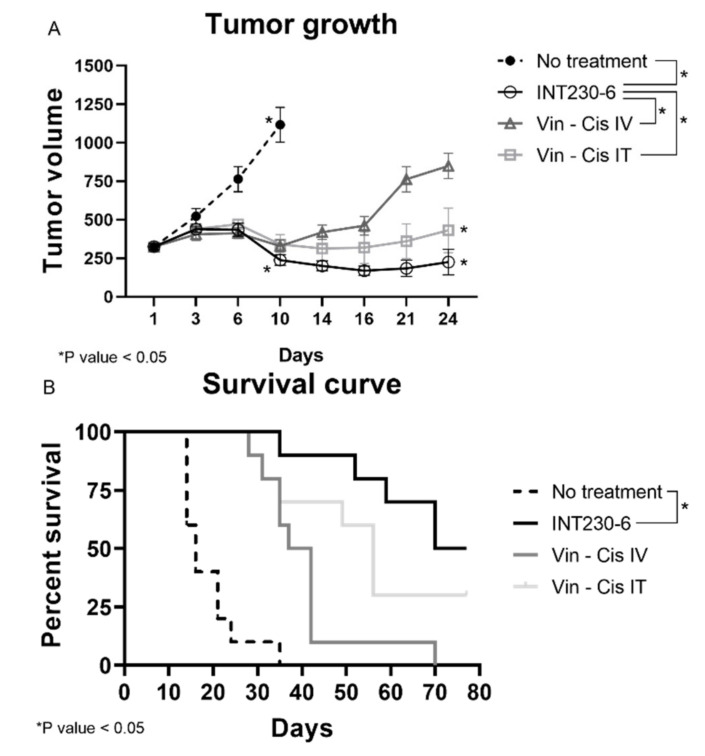
INT230-6 in vivo treatment of Colon-26 tumors Tumor Growth Inhibition. BALB/c female mice were inoculated with 1 × 10^6^ Colon-26 tumor cells in the right flank (cell injection volume, 0.1 mL/mouse). A total of 10 mice were assessed in each group and treated with intratumoral (IT) doses of INT230-6 or IT Vinblastine + Cisplatin or IV doses of Vinblastine + Cisplatin when mean tumor volume reached 325 mm^3^ (dose was 100 µL/400 mm^3^ tumor volume). Cisplatin was administered at 0.5 mg/mL while Vinblastine at 0.1 mg/mL once a day for 5 consecutive days (QDx5). (**A**) Tumor growth curves represented by the mean tumor volume of each group and error bars represent the SEM. Asterisks are representative of *p* values < 0.05 in the group’s comparison calculated with two-way ANOVA. (**B**) Kaplan–Meier survival curves of the Colon-26 tumor bearing mice. Asterisks are representative of *p* values < 0.05 in the group’s comparison and calculated through Log-Rank (Mantel-Cox) Test.

Having established that SHAO amphiphilic nature enhances drug’s dispersion throughout murine tumors, the tumor growth inhibition of drug formulations with and without enhancers was then assessed in vivo. For this purpose, INT230-6, was tested in large Colon26 tumor models in Bagg albino, strain c (BALB/c) mice. In these studies, untreated tumors grew rapidly and approximately 90% of untreated control animals needed to be euthanized or died in three weeks. Tumors in mice receiving INT230-6, however, showed decreased mean tumor size. In addition, INT230-6 treatment showed improved survival when compared to animals receiving cisplatin and vinblastine alone (IV or IT) (Figure 3A).

Treatments initiated when the baseline mean tumor volume was approximately 325 mm^3^. The INT230-6-treated group tumors regressed to a mean value of 238 mm^3^ at Day 10 and remained below baseline (296 mm^3^) until Day 31, which corresponded to the day of the first death in the group. In this experiment, animals in another group received the same drugs IT at the same dose and concentration without the enhancer in the formulation. This group had a mean tumor volume increase ranging from 323 mm^3^ at baseline to 340 mm^3^ at Day 10 and 432 mm^3^ at Day 24 (corresponding to the day prior to the first death in that group). Similarly, animals in the group with the drugs given as an IV formulation at the same drug dose and concentration as INT230-6 (without the cell penetration enhancer) had a mean tumor volume ranging from 324 mm^3^ at baseline to 329 mm^3^ at Day 10 and 849 mm^3^ at Day 24 (the day of the first death in that group). Statistical assessment of tumor growth inhibition in the Colon-26 murine tumor model found that INT230-6 was significantly superior in regressing tumors to the drugs given IV (*p* = < 0.0001) and IT (*p* = 0.023).

In addition, the Kaplan–Meier estimation was used to assess the survival of the mice (Figure 3B). The median overall survival was 16, 37, 52 and 77, days for no treatment, drugs administered alone IV, drugs administered alone IT, and INT230-6 IT, respectively. Two animals receiving the INT230-6 formulation had a complete response (CR). Overall, these data show that SHAO, when co-formulated with cisplatin and vinblastine (INT230-6) and delivered intratumorally, improves tumor growth inhibition over the drugs alone given IT or IV.

### 2.3. Colon26 Complete Responders Treated with INT230-6 Show Immunity upon Re-Challenge

**Figure 4:**
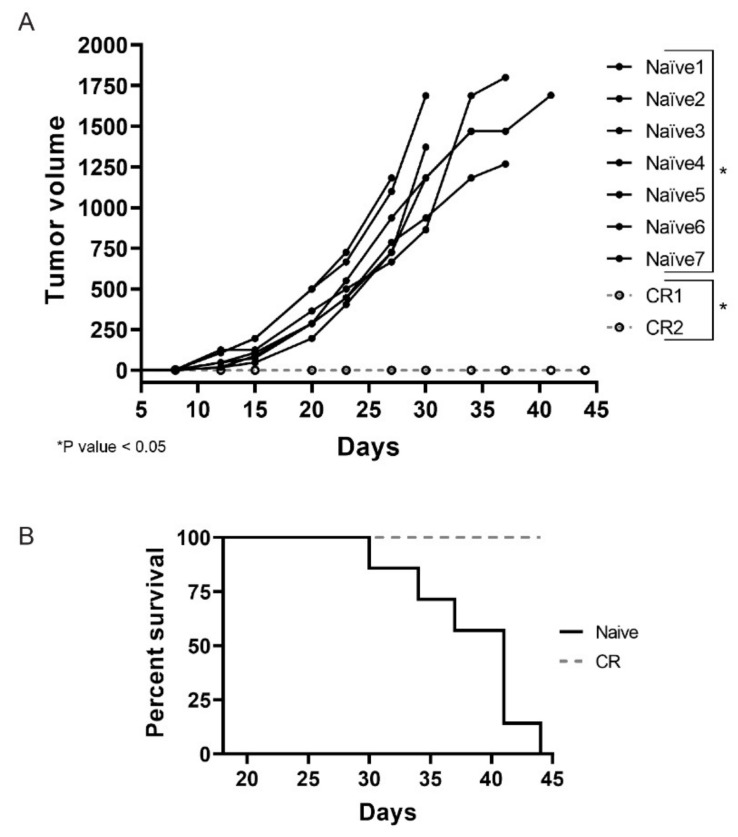
INT230-6 complete responders show immunological memory upon re-challenge. (**A**) The individual tumor growth curves of seven naïve BALB/c mice and the two complete responders (CRs) previously treated with INT230-6, inoculated with 1 × 10^6^ Colon-26 tumor cells in the flank opposite to original inoculation. The two groups show statistical differences (*p* value < 0.05) calculated with two-way ANOVA on the mean tumor volume of each group. (**B**) Kaplan–Meier curves assessing the survival of mice for 44 days. None of the CR mice showed tumor development for the entire length of the study. No treatment was administered in this study.

Necrosis is a feature commonly observed in INT230-6-treated tumors. These regressing lesions could offer the opportunity to elicit an immune response due to antigens released in the tumor microenvironment as a consequence of the cell death caused by active components of INT230-6. This becomes particularly relevant for those mice that showed a complete tumor remission after treatment. To test whether complete responder animals would be protected from developing tumors when re-challenged with the same tumor cells, CR animals from the INT230-6 group were reinoculated with Colon26 tumor cells in the contra-lateral side relative to the first inoculation site. Seven naïve mice were also inoculated as control. In Figure 4A, an individual tumor growth curve for each mouse are shown. Interestingly, all naïve mice developed tumors while none of the CR animals did. By the end of the study (day 45), all naïve mice died or needed to be euthanized, while 100% of the CR were still alive (Figure 4B). It is worth noting that, in this experiment, mice received no treatment and the lack of tumor development in the complete responders suggest that INT230-6 immunized the mice against Colon26 tumor cells.

### 2.4. Histological Assessment

**Figure 5:**
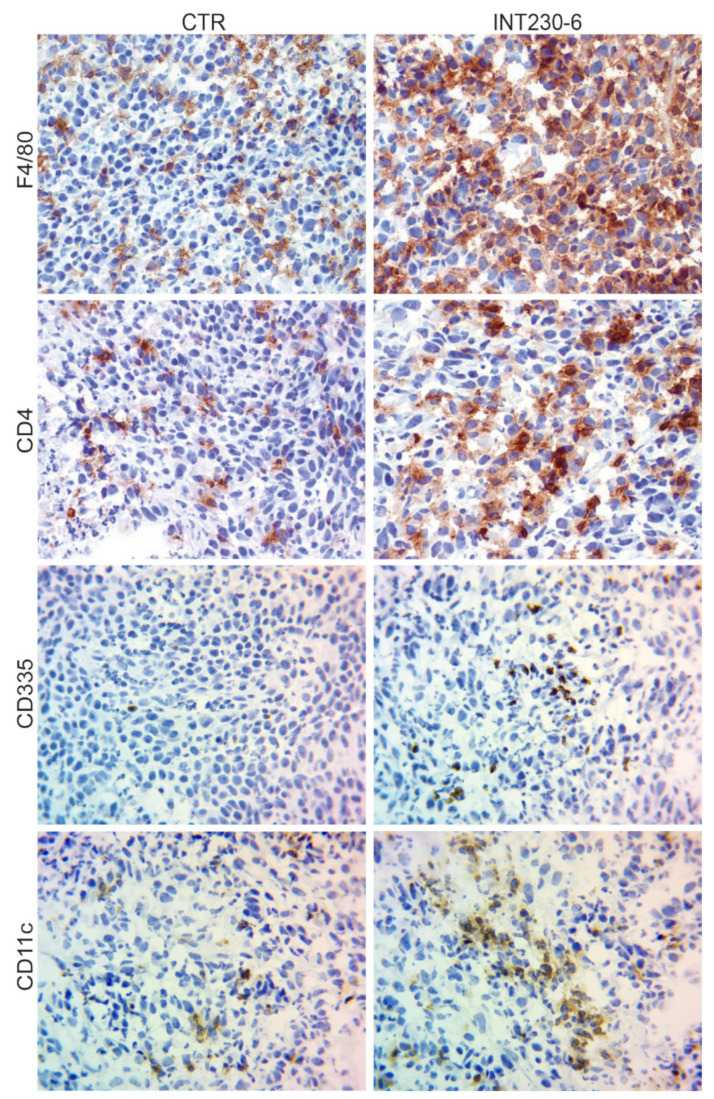
IHC analysis of Colon-26 tumors untreated and INT230-6-treated tumors ~10 days post dose.

The lack of tumor development in the CR mice shown in Figure 4, suggests that immune cells can infiltrate more efficiently and/or become more efficiently activated in the INT230-6-treated tumors. To assess the presence of immune markers in treated vs. untreated tumors, a histological tumor study was undertaken. Greater necrosis in INT230-6-treated tumors was observed compared to controls at three days post dose. INT230-6-treated tumors were 75% necrotic, whereas untreated tumors were less than 10% necrotic. For this study, random subsets of animals were sacrificed at various timepoints. It was not possible to determine which animals were responding to treatment prior to sacrifice. In comparing treated animals to non-treated controls, immunohistochemistry (IHC) analysis of immune cell markers was conducted. An increased staining for CD4+ (T-cells), F4/80 (macrophages), CD335 (natural killer cells), and CD11c (dendritic cells) was detected in treated animals (Figure 5). This is consistent with the hypothesis that INT230-6 enhances immune cell infiltration, possibly due to the higher number of epitopes available in the tumor, as a consequence of the localized cell death (necrosis) consistently observed. Enhanced immune cells infiltration was also observed in our recently published article [33], where CD8+ cells were observed in the tumor at different time points after treatment. INT230-6 IT and the capability of these cells in specifically killing Colon-26 cells was demonstrated in vitro.

Representative images (40× magnification) of IHC staining for CD4+ (T-cells), F4/80 (macrophages), CD335 (natural killer cells), and CD11c (dendritic cells). In general, the staining appeared more evident in the INT230-6-treated tumors, particularly toward the more necrotic areas.

Overall, these data show that the presence of the immune cells markers in tumors increased after IT delivery of INT230-6. Furthermore, the involvement of the immune cells after treatment is also supported by the inability of tumors to grow in animals having had a complete response upon re-challenge. More in depth immune infiltrating immune cell analysis on INT230-6 is reported (see Bloom [33])

### 2.5. Tumor Growth Inhibition and Survival in Murine 4T1 Tumors

**Figure 6:**
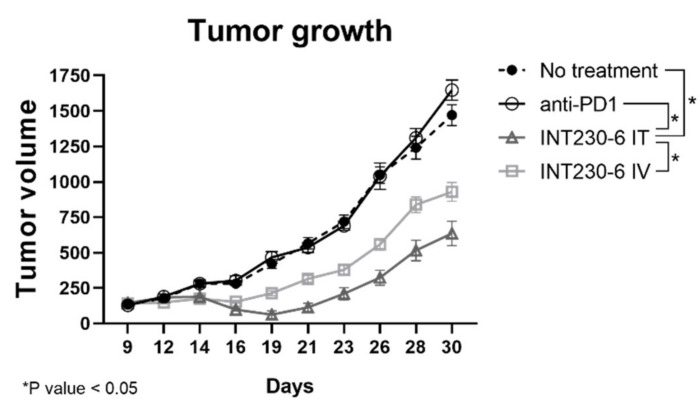
Tumor growth inhibition in 4T1 tumor bearing mice treated with INT230-6. Tumor growth curves showing BALB/c mice inoculated with 1 × 10^6^ 4T1 tumor cells in the right flank and treated with INT230-6 IT (QDx3), INT230-6 IV (QDx3), anti-PD-1 ((Q3Dx2; 3 off) x2) and untreated controls. Due to rapid metastasis formation, mice were randomized at approximately 125 mm^3^ mean tumor volume. Two mice from the INT230-6 IV group were removed from the study for weight loss. Asterisks represent statistically different groups (*p* value < 0.05, calculated with two-way ANOVA).

To assess whether INT230-6 would also be able to regress tumor growth in a different model, a second experiment with the 4T1 breast tumor cell line was performed. In this study, a group was treated with a murine anti-programmed cell death protein 1 (PD-1) antibody. INT230-6 regressed mean tumor volumes from baseline value of 143 to 62 mm^3^ at Day 19 (Figure 6). The untreated group had a mean tumor volume increase from 133 mm^3^ at baseline to 420 mm^3^ at Day 19 and the mean tumor volume of the anti-PD-1 group increased from 128 to 466 mm^3^ in the same period. After 30 days, while the no treatment and the PD-1 group reached a mean tumor volume of 1470 and 1647 mm^3^, respectively, the INT230-6 mice treated intratumorally exhibited a mean tumor volume of 635 mm^3^. Starting from day 19 until the end of the study, the INT230-6 IT tumor growth curve was significantly different from each other group (*p* value < 0.05, calculated with two-way ANOVA). Overall, these data show that INT230-6 reduces tumor growth in a second tumor model in addition to the Colon26 previously reported.

### 2.6. Combination of INT230-6 with Checkpoint Blockade

**Figure 7:**
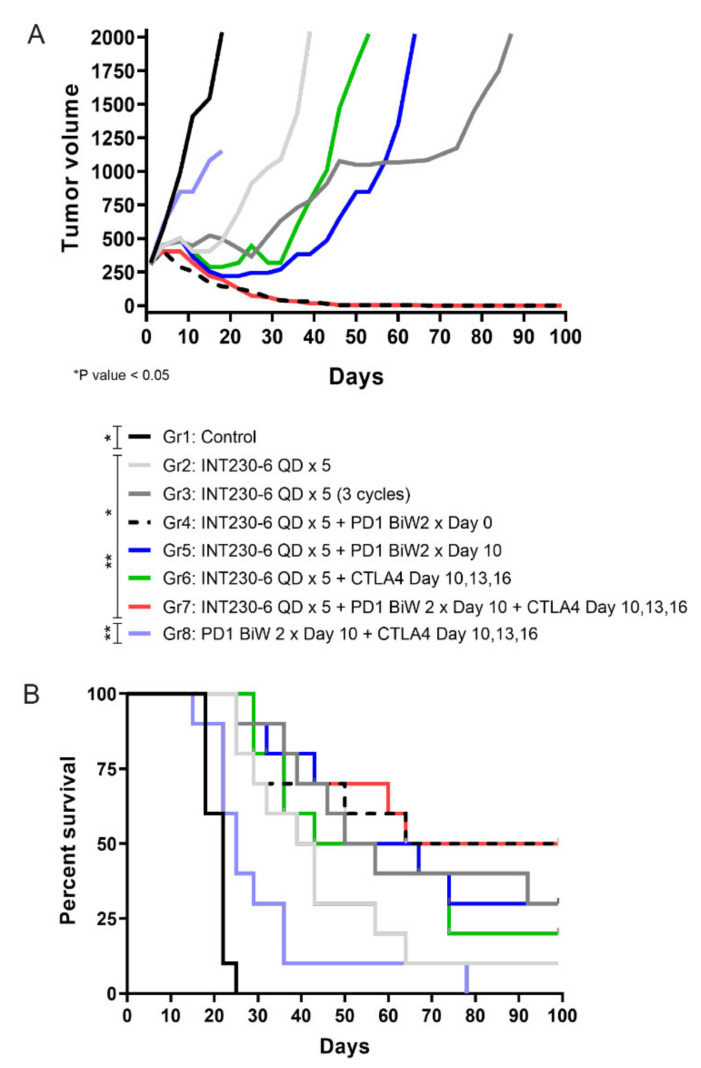
Effects of INT230-6 alone and in combination with checkpoint inhibitors (CTLA-4 and/or PD-1 antibodies) on median tumor volume in mice bearing Colon-26 tumors. (**A**) There were ten (10) animals per group. The median tumor volume was chosen to better represent the tumor growth curves of BALB/c mice for the full length of the study (100 days). The following animals were found dead in the cages and removed from the study for a better representation of the tumor growth curves: Group 1 (1 Animal), Group 2 (3 animals), Group 3 (2 animals), Group 4 (1 animal), Group 5 (1 animal), Group 6 (1 animal), Group 7 (1 animal), Group 8 (1 animal). Because mice died at different times during the study, statistical analysis was conducted with two-way ANOVA at day 22 (latest available data for the control group 1). At this time, INT230-6-treated groups had statistically significant reduced tumor growth curves when compared to either Group 1 (Control) or Group 8 (anti-PD-1 + anti-CTLA-4) (*p* value < 0.05). (**B**) Kaplan–Meier survival curves showing that the addition of anti-PD-1 antibodies to a single cycle (QDx5) of INT230-6 treatment, increased the number of CR animals from 1/7 (14%) (Group 2) to 5/9 (55%) (Group 4). A similar result was obtained in Group 7 (INT230-6 + anti-PD-1 + anti-CTLA-4), where 5/9 mice had CR (55%) considering that CTLA-4 alone added limited benefits in combination with INT230-6; 2/9 CR in Group 6 (22%). The log rank (Mantel-Cox) test showed statistical differences between groups treated with INT230-6 and Group 1 and Group 8.

Given the tumor immunity demonstrated post INT230-6 injection, an assessment was done to evaluate combinations of INT230-6 with immune checkpoint inhibitors. A study was conducted where intratumoral INT230-6 was given in conjunction with IV delivered anti-PD1 and/or anti-CTLA-4. The Colon26 cell line in larger size tumors (>200 mm^3^) is not highly responsive to treatments with PD-1 or CTLA-4 antibodies as monotherapy. Thus, the observation of therapeutic and immune activation benefit can be determined and understood when checkpoint drugs are combined with INT230-6 compared to dosing both IT INT230-6 and checkpoint drugs alone. All the INT230-6-treated groups had statistically significant reduced tumor growth compared to the untreated and the CTLA-4 + PD-1 group, where tumors continued to grow without any evidence of regression (Figure 7A). Interestingly while the INT230-6 monotherapy administered for a single 5-day cycle resulted in tumor regressions in all animals and a 14% CR rate (1/7), the three repeated INT230-6 cycles (5-day treatment followed by 9 days of rest), improved the CR rate to 37% (3/8). The INT230-6 single 5-day cycle, however, increased the number of CR to 55% (5/9) with concurrent administration of the PD-1 antibody and to 33% when PD-1 antibody was delayed until after INT230-6 treatment (3/9) (Figure 7B). The combination of CTLA-4, PD-1 and INT230-6 also increased the number of CR to 55% (5/9). No significant improvements were seen in the group treated with a CTLA-4 antibody and INT230-6 (22% CR) (2/9) compared to INT230-6 monotherapy in this single tumor model.

Mice receiving PD-1 plus CTLA-4 antibodies in the control group experienced notable morbidity (ulcerations, weight loss) indicative of nonspecific inflammation likely associated with broad immune activation. (This morbidity is similar to what is seen in greater than 50% of patients with melanoma who experience at least grade 3 toxicity with PD-1 and CTLA-4 antibody combination clinically and often require dose reductions or permanent discontinuations [34]). Of interest, these toxicities were not seen in mice when the same doses were given in combination with INT230-6. Large Colon26 tumors do not benefit from murine checkpoint inhibitors as monotherapy. Tumor ulceration and off target toxicities often result, which, indeed, was observed in Group 8. After INT230-6 treatment, however, tumor cell death releases antigens in the microenvironment to attract antigen presenting cells as shown in Figure 5. The directed immune cells enter into the tumor microenvironment and away from healthy tissue, thereby reducing the systemic toxicity.

## 3. Discussion

Direct IT therapy has been proposed as a means of improving the therapeutic index of active IV formulated drugs [9,10,11,12,13,14,15,16]. Unfortunately, the direct IT therapeutic strategies investigated to date have had limited success [8], primarily due to the lack of efficacy beyond the area injected. It is postulated that the lack of efficacy of IT approaches has been due to poor drug dispersion within the tumor, lack of absorption by the tumor coupled with ejection post injection. In addition, a limited penetration into the cancer cell, as well as the inability to affect distal sites of disease have also decreased the utility of past or current IT approaches. Through the use of a novel formulation chemistry, the research conducted in our studies has identified a potent tumor tissue dispersing formulation containing a fixed ratio of an amphiphilic cell penetration enhancer molecule, SHAO, formulated with cisplatin and vinblastine and designated as INT230-6. Improved drug absorption/dispersion using INT230-6 led to significantly greater tumor regression and increased survival benefit compared to the drugs alone given IV or IT. Using large tumors, INT230-6 results in a number of CRs in multiple tumor types. Furthermore, the results show that INT230-6 treatment increased tumor necrosis and the appearance of certain immune cell infiltrates in the tumor microenvironment. Indeed, the presence of tumor antigens released by the dying cells of a highly necrotic tumor appears to create an environment favorable to induce a systemic immune response. This hypothesis is supported by the re-challenge experiment showing that complete responders from previous INT230-6 treatments developed immunity against the same cell lines. Literature studies show that cisplatin has activity in multiple tumor types [35], and platinum agents have the ability to induce immunologic cell death in part by release of calreticulin [36]. Another possibility could be that the enhancer-based IT delivery modality may increase the calreticulin release, located in storage compartments associated with the endoplasmic reticulum, to the cell surface. Cisplatin can also result in high mobility group box 1 (HMGB1) protein production, thereby stimulating mature dendritic cell processing through interaction with toll-like receptor-4 (TLR-4) [36,37].

While repeat INT230-6 (cytotoxic) treatment to the same tumor in theory could impair or kill the beneficial immune cells recruited to the tumor microenvironment, the data generated indicate that repeated INT230-6 treatment yielded superior CR rates and overall survival compared to single treatment. This finding leads to a preferred clinical regimen of repeated INT230-6 cycles.

The current clinical success of checkpoint antibodies in many major tumor types is limited due to their inability to overcome T-cell suppression or improve antigen recognition. The combination of INT230-6 to attenuate tumors and improve antigen presentation for immune recognition given IT either alone or with checkpoint inhibitors may have promise for improved clinical anticancer activity. There is further experimental evidence in mice that the systemic administration of chemotherapeutic agents impairs the immune response to a PD-1 antibody, while the local administration of these potent agents potentiates immune activity [38]. In the clinic, Ariyan [39] reported that the isolated limb infusion of melphalan with systemic ipilimumab, an CTLA-4 antibody, in patients with in transit melanoma, showed a durable increase in efficacy over ipilimumab alone.

INT230-6 is currently undergoing phase 1/2 dose escalation in a clinical trial investigating repeating doses, dose frequency and drug load per tumor alone and in combination with a commercial PD-1 antibody (pembrolizumab) and a CTLA-4 antibody (ipilimumab) in several different refractory solid tumor cancers (NCT03058289). Patients will be followed for safety and will be evaluated for both injected and bystander tumor responses. The study also tracks the pharmacokinetics of each INT230-6 component. In addition to clinical endpoints, blood and tumor samples will be evaluated to look at the tumor microenvironment and central immune compartment. A better understanding of the tumor and its stroma will enable the customization of IT-designed formulations such as INT230-6 for improved patient outcomes.

## 4. Materials and Methods

Experiments included the evaluation of INT230-6 dispersion in tumors with and without the enhancers; the effect in vitro on cancer cell morphology; the evaluation of certain immunomodulatory effects of IT treatments, including immune cell infiltration into the tumor microenvironment; the growth inhibition, tumor regression and synergy of INT230-6 when combined with checkpoint inhibitors.

### 4.1. Formulation

**Figure 8:**
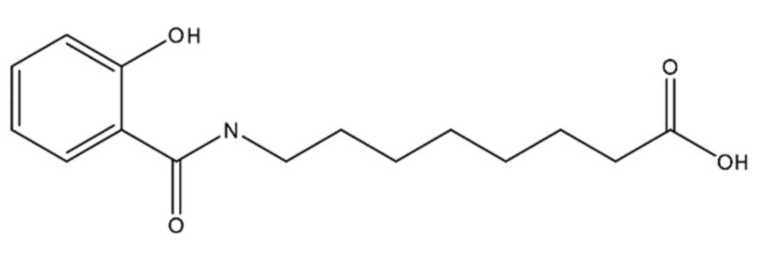
Molecular structure of 8-((2-hydroxybenzoyl)amino)octanoate, also referred to as SHAO.

The penetration enhancer molecule in INT230-6 is a 279 molecular weight agent, 8-((2-hydroxybenzoyl)amino)octanoate (referred to as SHAO in solution and obtained from AMRI Global Albany, New York USA), and is considered an excipient with no pharmacologic activity of its own. The compound’s molecular formula is C_15_H_21_NO_4_ and the structure is shown in Figure 8. The two active pharmaceutical ingredients in the INT230-6 formulation are cisplatin (CAS ID15663-27-1) and vinblastine sulfate (CAS ID1143-67-9) both obtained from Tocris Bioscience a division of Bio-Techne Corporation, Minneapolis, MN USA. The composition of INT230-6 is 10 mg/mL of SHAO, 0.5 mg/mL of cisplatin, and 0.1 mg/mL of vinblastine. To prepare INT230-6, the desired amount of SHAO was dissolved in a ~0.35 M NaOH solution, followed by the addition of 0.1% Tween80 (Cat No. AC278632500, Fisher scientific, Waltham, MA USA) and the required amount of the active agents, and then filtered for sterilized dosing.

### 4.2. Cell Lines

Murine cell lines included 4T1 breast cancer cells obtained from the American Type Culture Collection (Manassas, VA), human BxPc3-luc2 pancreatic adenocarcinoma cells obtained from Caliper Life Sciences (Hopkinton, MA USA), and Colon-26 murine adenocarcinoma cells obtained from the National Cancer Institute, Bethesda, MD USA.

### 4.3. Animals

All experiments were approved by the US Public Health Service Policy on Humane Care and Use of Laboratory Animals and carried out either at the MI Bioresearch division of Covance / LabCorp in Ann Arbor Michigan USA, CR Discovery Services, Morrisville, NC USA (a division of Charles River Laboratories (CRL), or Taconic Biosciences Hudson, NY USA. Female mice (BALB/c or Nude severely compromised immune deficient mice, obtained from Charles River Laboratories Wilmington, MA, USA, aged 6 to 12 weeks were used. MI BioResearch, Taconic Biosciences and CR Discovery Services specifically comply with the recommendations of the Guide for Care and Use of Laboratory Animals with respect to restraint, husbandry, surgical procedures, feed and fluid regulation, and veterinary care (Supplementary Materials Text S1).

Animal health and behavior were monitored twice per day. Any individual animal with a single observation of >30% body weight loss or three consecutive measurements of >25% body weight loss was euthanized.

The endpoint of the experiment was a tumor volume of 2000 mm^3^. When the endpoint was reached, the animals were to be euthanized immediately.

The animals were terminated by CO2 asphyxiation or cardiac puncture depending on the lab and study. This was followed by cervical dislocation. All studies were approved by the specific site’s Institutional Animal Care and Use Committee (IACUC). Experiments were performed at multiple locations (Supplementary Materials Text S2).

### 4.4. Drug/Enhancer Dispersion Studies in Murine Models

Drug dispersion in tumors was assessed in three studies at three laboratories in female nude mice bearing BxPc3-luc2 pancreatic tumors. In one experiment, mice approximately 5 to 6 weeks of age were implanted with cryopreserved BxPC3 fragments (MI3319) The site’s Institutional Animal Care and Use Committee (IACUC) approvals were approximately March 2018 and July 2018. When tumors reach ~750 mm^3^, the fragments were passed to additional animals and then those additional animals were passed fragments until a sufficient number of animals had tumors on their right flank of sufficient size to conduct the study. Three drops (~150 µL) of India Ink was added to a 10-mL vial of INT230-6 clinical supplies grade drug product. A control solution of 10 mL using the same aqueous vehicle with 0.1% Tween80, cisplatin (0.5 mg/mL) and 150 µL of India Ink was also prepared. Eight animals were dosed with INT230-6/Ink solution and six animals were dosed with drug/Ink alone. All animals were oriented in the same position for dosing. A 26-gauge needle with a butterfly valve was placed in the center of the tumor at the same angle for each of the injections. IT dosing was performed over 90 s using a dosing pump approximately 0.75 cm from the tumor surface. A drug volume of 0.075 or 0.225 mL was administered at tumor volume ratios of approximately 1:11 and 1:4. Animals were sacrificed, necropsied and examined for solution in the abdominal and chest cavities. All tumors were excised, split in half along the same axis and examined immediately following dose completion. Direct measurement and observations on Ink containing solution diffusion diameters and ex-tumor leakage were made.

### 4.5. Tumor Growth Inhibition and Survival

Growth inhibition studies assessed the pharmacodynamic effects of INT230-6 in in vivo murine models. The effect of INT230-6 on tumor growth inhibition and survival was assessed in mice bearing large tumors in the hind flank compared to controls (i.e., no treatment, enhancer alone, or drugs alone dosed IV and/or IT). Abnormal behavior (e.g., food consumption, body weight, activity levels) was assessed as a measure of toxicity. Tumor volume was calculated by caliper using the measured width squared (w2) in millimeters (mm) multiplied by the length (mm) divided by 2. The volume (V) equation was V = (w2 × L)/2. Tumor size and animal survival were assessed over time, and animals were terminated per protocol using the methods described in S2 Text once the tumor volume was above 2000 mm^3^. A complete response (CR) was defined as tumors completely disappearing with no measurable caliper areas.

### 4.6. Colon-26 Studies

Colon-26 was chosen as the primary cell line for the first set of growth studies as this murine cancer type is commonly used to test agents in syngeneic animals. Larger initial tumor volumes represent advanced disease and are a much more challenging model to demonstrate tumor regression or increased survival. In general, for Colon-26 studies, BALB/c female mice were injected in their right flank with 1 × 10^6^ Colon-26 tumor cells (cell injection volume, 0.1 mL/mouse). Animals were treated when the mean tumor volume reached 300 mm^3^ after randomization and only mice with tumors smaller than 500 mm^3^ were enrolled in the study. The mean standard deviation of the groups was approximately 16 to 17 mm^3^. Randomization typically occurred 17 to 19 days following cancer cell inoculation. Mice receiving IT treatment were administered 0.1 mL per 400 mm^3^ tumor volume for 3 to 5 days depending on the study. IT and IV drug alone doses were comparable in total drug dosed. Experiments in the series were Colon E230 and E236 with IACUC approvals made approximately on August 2013 and December 2013.

### 4.7. 4T1 Studies

To confirm the anti-cancer activity of INT230-6, growth inhibition studies were conducted in a second cell line, 4T1. Due to the rapid formation of metastases, mean tumor volumes for 4T1 studies were approximately 125 mm^3^ after animal randomization into test groups. BALB/c female mice were injected in their right flank with 1 × 10^6^ cells. The cell implant volume was 50 µL and randomization occurred 7 to 9 days following cancer cell inoculation. Mice receiving IT treatment were administered 0.1 mL per 400 mm^3^ tumor volume for 3 to 5 days depending on the study. In one study, INT230-6 was dosed once daily (QD) for three days (QDx3) at 0.1 mL IT, which contained at total 50 µg of cisplatin and 10 µg of vinblastine. Anti-mPD1 (RPM1-14, cat# 5792-599016j1, BioXCell, Lebanon, NH USA) antibodies were dosed in two cycles of three consecutive days and 3 days off (Q3Dx2; 3 off) x2. The experiment reported in 2.5 was MI2477 with IACUC approval made approximately on January 2016.

### 4.8. In Vitro Cell Membrane Retention Studies

To determine whether the enhancer does not disrupt to the cancer cell membrane, in vitro studies were conducted investigating SHAO alone in a static system. Solutions of the enhancer at various concentrations together with a control were incubated for up to 24 h with the Colon-26 cell line. Photomicrographs showing the morphology of the cells were taken.

### 4.9. Checkpoint Combination Studies

There is potential for an increased response of checkpoint inhibitors using INT230-6 due to the likely presentation of antigen and recruitment of T-cells following cell death. Studies using IT dosed INT230-6 concurrently or sequentially with IV administered monoclonal antibodies against cytotoxic T-lymphocyte antigen 4 (CTLA-4) (9H10, lot# 5294/0814, BioXCell, Lebanon, NH USA) and/or programmed death-1 (PD-1) (RPM1-14, lot# 5311-4/0714C, BioXCell, Lebanon, NH USA) were conducted. BALB/c female mice aged 8 to 12 weeks were injected in their right flank with 1 × 10^6^ Colon-26 tumor cells using a cell injection volume of 0.1 mL/mouse at Charles River Laboratories (CRL). INT230-6 was administered IT to mice with tumors approximately 300 mm^3^ in size; CTLA-4 and PD-1 antibodies were administered IV. In one study, the following groups were assessed: no treatment (Group 1; control); INT230-6 administered IT once daily for 5 days (Group 2); INT230-6 administered IT once daily for 5 days (with 9 days off) for 3-dose cycles (Group 3); INT230-6 administered IT once daily for 5 days and PD-1 antibody administered twice weekly for 2 weeks beginning on Day 0 (Group 4); INT230-6 administered IT once daily for 5 days and PD-1 antibody administered twice weekly for 2 weeks beginning on Day 10 (Group 5); INT230-6 administered once daily for 5 days and CTLA-4 antibody administered on Days 10, 13, and 16 (Group 6); INT230-6 administered once daily for 5 days, PD-1 antibody twice weekly for 2 weeks beginning on Day 10, and CTLA-4 antibody on Days 10, 13, and 16 (Group 7); and PD-1 antibody administered twice weekly for 2 weeks beginning on Day 10 and CTLA-4 antibody administered on Days 10, 13, and 16 (Group 8; control). Mice were observed frequently for health and overt signs of any adverse treatment related side effects and noteworthy clinical observations. Considering the length of the study (100 days), the mice occasionally found dead in the cages and not reaching 2000 mm^3^ were removed from the study for a better representation of the tumor growth curves. The list of removed mice is as follows: Group 1 (1 animal), Group 2 (3 animals), Group 3 (2 animals), Group 4 (1 animal), Group 5 (1 animal), Group 6 (1 animal), Group 7 (1 animal), Group 8 (1 animal). 

### 4.10. Immune Cell Flux Immunohistochemistry Evaluations

The tumors from two groups of 12 animals (INT230-6 treated and untreated) were evaluated histologically for immune cell markers (CD4, F4/80, CD11c, and CD335). Each animal was inoculated with 1 × 10^6^ Colon-26 colon cancer cells on one flank. When group tumors reached a mean value of 323 mm^3^ per group, treated animals received daily IT injections of INT230-6 for five consecutive days. At six timepoints over a period of 10 days, two animals from each group had their tumors excised, the amount of necrosis estimated, and the tissue fixed and shipped to CRL (Fredericksburg, MD, USA) for immunohistochemical evaluation. Immunohistochemical staining was performed to detect various immune cell markers (CD4, F4/80, CD11c, and CD335) to evaluate the presence of infiltrating mononuclear cell populations within the tumor. To detect the markers within the test tissues, the detection antibodies were applied to acetone/formalin-fixed (CD335) or formalin fixed (CD4, F4/80, and CD11c) cryosections of the tumors. Blocking buffer composition was PBS + 1% bovine serum albumin (BSA); 0.5% casein; and 1.5% normal donkey serum. Following the protein block, the primary antibodies (Rat anti-mouse CD4 (cat# 553043 BD Pharmingen, Woburn, MA USA), Rat anti-mouse F4/80 ( cat# MCA497R, BioRad Kidlington, UK formerly AbD Serotec) and Rat IgG2a, kappa (cat# 559073, BD Pharmingen, Woburn, MA USA) (1 μg/mL for 1 h), or none (buffer alone as the assay control)) were applied to the slides at a concentration of 1 μg/mL for one hour. Hamster anti-mouse CD11c (cat# MCA1369GA, AbD Serotec, Hercules, CA USA), Hamster IgG (cat# 007-000-003, Jackson ImmunoResearch West Grove, PA USA), or none (buffer alone as the assay control) was applied to the slides at a concentration of 1 μg/mL for one hour. Rat anti-mouse CD355 (cat# 137602, BioLegend Dedham, MA USA), Rat IgG2a, kappa (cat# 559073, BD Pharmingen, Woburn, MA USA), or none (buffer alone as the assay control) was applied to the slides at a concentration of 10 μg/mL for one hour.

Experiments in the series for sections 4.09 and 4.10 were CR-E262, CRL-E241, IACUC approvals made approximately November 2014 and July 2014.

## 5. Conclusions

The data generated suggest that IT use of a formulation containing the amphiphilic molecule SHAO increase drug dispersion and cell penetration without membrane disruption. INT230-6, currently in clinical testing, containing such a molecule formulated with two potent cytotoxic agents, increases tumor kill for multiple murine tumor types. The rapid attenuation of a sufficient mass of cancer cells in their three-dimensional tumor microenvironment appears to increase the presence of immune cells and potentially primes a systemic immune response against the cancer. Furthermore, the INT230-6 formulation appears synergistic with checkpoint antibodies. These murine results indicate potential for INT230-6 to be a highly effective anti-cancer treatment alone or in combination with a checkpoint inhibitors.

## 6. Patents

The product described in this article, INT230-6, is part of several US and Foreign patents granted to Intensity Therapeutics, Inc. In the US, the patents issued to date are 9,351,997 issued on May 31, 2016, and 9,636,406 issued on May 2, 2017.

## Supplementary Materials

Supplementary materials can be found at https://www.mdpi.com/1422-0067/21/12/4493/s1. **S1 Text. Animal Care:** The animal care and use program at Taconic Biosciences employs the International Health Monitoring System (IHMS™), which is defined by a comprehensive list of microbiological agents, test frequencies, and test methods, and meets or exceeds Federation of European Laboratory Animal Science Associations (FELASA) guidelines. Taconic complies with Sthe guide for the care and use of laboratory animals. CRL systems conform and are maintained according to the NIH standards established in the Guide for the Care and use of Laboratory Animals and CR Discovery Services are accredited by the Association for Assessment and Accreditation of Laboratory Animal Care International, which assures compliance with accepted standards for the care and use of laboratory animals. **S2 Text. Experimental Locations:** Dispersion and diffusion experiments in nude mice were performed at multiple sites; Taconic Biosciences (Cranbury, NJ, USA), MI Bioresearch (MIB, Ann Arbor, MI, USA) and CRL (Morrisville, NC, USA). Colon-26 and 4T1 in vivo studies in BALB/c mice were performed at both Charles River Laboratories (CRL Morrisville, NC, USA) and MI BioResearch. Staining, tissue preparation, and slide reading for immunohistochemistry from CRL conducted at the Charles River Laboratory’s Fredericksburg, MD site or MIB.
