# Supplementary material for: Intratumoral Administration of a Novel Cytotoxic Formulation with Strong Tissue Dispersive Properties Regresses Tumor Growth and Elicits Systemic Adaptive Immunity in In Vivo Models
Source: Int J Mol Sci. 2020 Jun 24;21(12):4493. doi: 10.3390/ijms21124493 (PMC7349938; doi:10.3390/ijms21124493)
Supplement: Supplementary file 1 [file ijms-21-04493-s001.pdf]

Supporting information

S1 Text. Animal Care. The animal care and use program at Taconic Biosciences employs the International Health Monitoring System (IHMS™), which is defined by a comprehensive list of microbiological agents, test frequencies, and test methods, and meets or exceeds Federation of European Laboratory Animal Science Associations (FELASA) guidelines. Taconic complies with the guide for the care and use of laboratory animals. CRL systems conform and are maintained according to the NIH standards established in the Guide for the Care and use of Laboratory Animals and CR Discovery Services are accredited by the Association for Assessment and Accreditation of Laboratory Animal Care International, which assures compliance with accepted standards for the care and use of laboratory animals.

S2 Text. Experimental Locations. Dispersion and diffusion experiments in nude mice were performed at multiple sites; Taconic Biosciences (Cranbury, NJ), MI Bioresearch (MIB, Ann Arbor, MI) and CRL (Morrisville, NC). Colon-26 and 4T1 in vivo studies in BALB/cAnNCrl mice were performed at both Charles River Laboratories (CRL Morrisville, NC) and MI BioResearch. Staining, tissue preparation, and slide reading for immunohistochemistry from CRL conducted at the Charles River Laboratory's Fredericksburg, MD site or MIB.

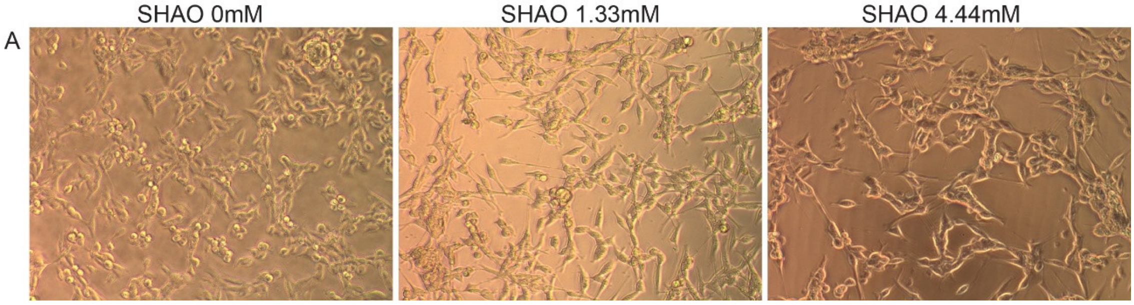

Figure 1. In vitro incubation showing cell morphology in presence or absence of SHAO molecule. Images showing 24h incubation in vitro of Colon-26 cells with SHAO: 0 mM (A), 1.32 mM (B) and 4.44 mM (C).
